# Factors Associated with Time to Sarcoma Diagnosis: Results from a Global Cross-Sectional Survey by the Sarcoma Patient Advocacy Global Network (SPAGN)

**DOI:** 10.3390/cancers18081256

**Published:** 2026-04-16

**Authors:** Verena Loidl, Kathrin Schuster, Roger Wilson, Denise K. Reinke, Ornella Gonzato, Matthew Sha, Lauren Pretorius, Vandana Gupta, Gerard Van Oortmerssen, Bernd Kasper

**Affiliations:** 1 Sarcoma Patient Advocacy Global Network (SPAGN), 61200 Woelfersheim, Germany; vloidl@ibe.med.uni-muenchen.de (V.L.); roger.wilson@sarcoma-patients.org (R.W.); dreinke@med.umich.edu (D.K.R.); vandana@vcarecancer.org (V.G.); g.van.oortmerssen@liacs.leidenuniv.nl (G.V.O.); 2 Institute for Medical Information Processing, Biometry, and Epidemiology (IBE), Faculty of Medicine, Ludwig-Maximilians-Universität München (LMU), 80539 Munich, Germany; 3 Department of Orthopaedics and Sports Orthopaedics, TUM University Hospital, Technical University of Munich, 81675 Munich, Germany; 4 Division of Hematology/Oncology, Department of Internal Medicine, University of Michigan, Ann Arbor, MI 48109, USA; 5 Paola Gonzato-Rete Sarcoma Foundation, 33100 Udine, Italy; 6 Faculty of Medicine, University of British Columbia, Vancouver, BC V6T 1Z3, Canada; mattsha@student.ubc.ca; 7 Campaigning for Cancer, Craighall, Johannesburg 2196, South Africa; lauren@campaign4cancer.co.za; 8 Mannheim Cancer Center, Mannheim University Medical Center, University of Heidelberg, 68167 Mannheim, Germany; bernd.kasper@umm.de

**Keywords:** sarcoma, sarcoma diagnosis, cancer diagnosis, time to diagnosis, diagnostic interval, diagnostic pathway, patient interval, care interval, misdiagnosis, referral pathway

## Abstract

**Background/Objectives:** Sarcomas are rare, highly heterogeneous malignancies comprising more than 100 subtypes. Their nonspecific clinical presentations, frequent misdiagnosis, and limited access to specialized expertise contribute to prolonged routes to diagnosis. Prolonged diagnostic intervals are associated with worse clinical outcomes, yet determinants of time to diagnosis remain insufficiently characterized. Therefore, the aim of this study was to examine clinical, health system, and socioeconomic determinants of time to diagnosis among patients with sarcoma. **Methods:** We conducted a global cross-sectional survey through the Sarcoma Patient Advocacy Global Network (SPAGN), including 1803 patients and caregivers across 19 languages. Collected data included sociodemographic characteristics, tumor-related factors, healthcare access, and diagnostic timelines. Associations between patient-, tumor-, and system-level variables and time to diagnosis were assessed using multivariable linear regression. Missing data were handled using multiple imputation. **Results:** The median time to diagnosis was 92 days (interquartile range (IQR): 31–245). Patient-reported barriers to care were strongly associated with prolonged diagnostic intervals. Female gender was independently associated with longer care intervals, while patients from low/lower-middle-income countries reported shorter diagnostic times. Tumor characteristics, age, and stage at diagnosis were not independently associated with time to diagnosis. **Conclusions:** Timely and accurate sarcoma diagnosis remains a global challenge. Prolonged routes to diagnosis appear to be driven by modifiable structural and socioeconomic factors, including misdiagnosis, limited specialist expertise, and unclear referral pathways, rather than tumor-related characteristics. These findings highlight the need to enhance awareness, streamline referral processes, and expand access to specialized care to reduce avoidable prolonged routes to diagnosis and improve patient outcomes worldwide.

## Simple Summary

Sarcomas are rare cancers that can arise in bones or soft tissues, and they are often difficult to diagnose because symptoms can be nonspecific and overlap with more common conditions. These characteristics are associated with prolonged time to diagnosis. When a sarcoma diagnosis occurs later, patients often report larger tumors and more complex treatment experiences. In this study, we asked more than 1800 patients and caregivers around the world about their experience getting a sarcoma diagnosis, aiming to explore factors contributing to prolonged routes to diagnosis. Prolonged routes to diagnosis were most frequently associated with healthcare system and social factors, including misdiagnosis, limited access to specialists, and unclear referral pathways, whereas sarcoma type or disease stage appeared less strongly associated with prolonged routes to diagnosis. These findings highlight that improving awareness of sarcoma, streamlining referral processes, and expanding access to specialized care could help patients receive faster and more accurate diagnoses, and potentially improve their outcomes.

## 1. Introduction

Sarcomas are rare and heterogeneous malignant tumors arising from mesenchymal tissues, including soft tissue and bone. They comprise more than 100 distinct histologic subtypes with diverse genetic, morphological, and clinical characteristics, collectively accounting for less than 1% of all adult malignancies and approximately 15% of pediatric cancers [1,2,3]. This histologic and molecular diversity, as highlighted in the 2020 WHO classifications, has led to major refinements in diagnostic terminology and subclassification [4].

Due to their rarity, heterogeneity, and nonspecific symptom presentation, sarcomas are frequently misinterpreted as benign or more common diseases, leading to substantial prolonged routes to diagnosis [5]. Misdiagnosis, diagnostic uncertainty, and changes in diagnosis along the care pathway are well-documented, with specialized sarcoma centers often correcting initial or inappropriate diagnoses [6,7,8]. Prolonged diagnostic intervals remain a persistent clinical challenge, despite the availability of advanced imaging and biopsy methods [5,9,10].

These prolonged routes to diagnosis are clinically relevant: long symptom intervals are associated with larger tumor size at presentation, increased treatment complexity, and poorer outcomes in terms of survival and quality of life [5,10,11,12]. Despite major technological and molecular advancements, the time to definitive diagnosis has remained largely unchanged over the past decades [9]. Moreover, survival for rare cancers, including sarcomas, remains substantially lower compared with more common malignancies and declines sharply with age [3,13].

Consequently, early recognition, accurate diagnosis and timely referral to specialized multidisciplinary centers are now considered critical to optimize patient outcomes and minimize the detrimental impact of prolonged routes to diagnosis [8,14]. Just as crucial for accurate and timely diagnosis is access to sarcoma expertise and specialists. However, this level of care is not available in many parts of the world, where multidisciplinary sarcoma expertise remains limited [15]. Although prolonged routes to diagnosis in sarcoma has been frequently reported, systematic data on its duration and the factors contributing to it remain limited and the variability in diagnostic trajectories complicates the analysis and interpretation of diagnostic time intervals [16]. Most existing evidence arises from retrospective, single-center studies that only provide a rough estimation of the time from symptom onset to diagnosis, without distinguishing specific phases of the diagnostic process.

The concept of the total diagnostic interval (TDI)—which covers the entire trajectory from first symptom to definitive diagnosis—provides a useful framework for analyzing diagnostic timeliness more precisely. Subdividing the TDI into the patient interval (PI) and the care or specialist interval (SI) helps identify sources of prolonged routes to diagnosis and potential targets for intervention [6,17,18]. A detailed examination of these intervals may facilitate the development of tailored strategies to shorten the diagnostic pathway and improve the efficiency and timeliness of sarcoma care.

Moreover, evidence remains limited regarding which patient-, tumor- or healthcare-related factors are associated with prolonged diagnostic intervals and which diagnostic steps contribute most to prolonged routes to diagnosis. A better understanding of diagnostic pathways and the factors involved could help identify at-risk patient groups, optimize referral processes, and ultimately enhance early detection and outcomes in sarcoma care.

Therefore, the aim of this study was to examine associations between patient- and tumor-related characteristics, education level, country income group, patient-reported barriers, and the time to diagnosis among patients with sarcoma.

## 2. Materials and Methods

### 2.1. Study Design and Setting

This international, cross-sectional study was administered online between 1 July and 31 December 2024. Sarcoma patients as well as caregivers of sarcoma patients, who had provided informed consent, were included. The study was open to all ages; caregivers could complete the survey on behalf of pediatric or incapacitated patients. Eligibility was independent of disease status, time since diagnosis, geographic location, healthcare system, treatment center, or histological sarcoma subtype.

### 2.2. Participant Recruitment and Consent

Participants were approached via SPAGN and its member organizations, sarcoma patient advocacy groups, professional networks, and online platforms. Informed consent was obtained electronically prior to survey completion. Caregivers were instructed to complete the survey only if patients were unable to do so, based on their knowledge of the patient’s diagnostic trajectory.

### 2.3. Survey Development and Pilot Testing

The survey collected patient- or caregiver-reported data on sociodemographics, tumor-related factors, and the diagnostic pathway including the timing of symptom onset, first healthcare consultation, diagnostic procedures, and final diagnosis (Supplementary Material S1). The questionnaire underwent pilot testing for completeness, clarity, and cultural appropriateness within the SPAGN. Medical accuracy and clinical relevance were reviewed by a sarcoma specialist/medical oncologist. Details on pilot materials are provided in Supplementary Material S2. Feedback informed iterative revisions of the survey in line with recommendations from the COSMIN initiative [19]. Translations into 19 languages followed a forward-backward procedure and were reviewed by native speakers familiar with sarcoma care.

### 2.4. Survey Administration and Data Collection Period

The survey was self-administered online, and data collection occurred from July to December 2024. A single questionnaire was used for both patients and caregivers, with wording adapted to respondent type (direct for patients, proxy-reported for caregivers). Respondent type was recorded at survey entry. Data refer to the patient, irrespective of respondent. Caregivers were instructed to complete the survey on behalf of the patient only if the patient was unable to do so (e.g., due to age, illness, or death), based on their knowledge of the diagnostic trajectory.

### 2.5. Variables

Primary Outcome: The primary outcome was time to diagnosis (days), defined as the interval between first symptom onset and confirmed sarcoma diagnosis. Symptom onset was based on patient- or caregiver-reported first noticeable symptoms, and the date of diagnosis referred to the time of clinical and/or histopathological confirmation, as reported by the participant.

Covariates: Sociodemographic variables included age (continuous, in years), gender (male/female/other), educational attainment, and country income group. Education was harmonized into six categories: no formal education, secondary education, vocational qualification, bachelor’s degree, advanced university degree, and other/unspecified. Country income level was classified according to World Bank categories and collapsed into low/lower middle, upper middle, and high income. Sarcoma subtype was categorized into major clinical groups (bone sarcoma, soft tissue sarcoma, and gastrointestinal stromal tumors). Primary tumor location was classified into clinically relevant anatomical regions. Stage at diagnosis was categorized as localized, advanced/metastatic, or unknown. Presence of a genetic syndrome was classified as yes, no, or unknown. Healthcare-related variables included initial contact at a specialized sarcoma center (yes/no), healthcare accessibility and affordability, and patient-reported diagnostic barriers, including difficulty finding a provider, long waiting times, misdiagnosis, lack of provider or patient awareness, and financial barriers. Presence of symptoms prior to diagnosis was dichotomized as yes/no.

### 2.6. Statistical Analysis

Descriptive statistics were summarized using medians and the interquartile range (IQR) for continuous variables and frequencies with percentages for categorical variables. Characteristics were reported for the full cohort.

We used multivariable linear regression to assess factors associated with time to diagnosis (in days) using a complete-case approach. The outcome showed a pronounced right skew, with a small number of zero values. To improve normality and allow inclusion of zero values, the outcome was transformed using a log(x + 1) transformation. Covariates were selected a priori based on clinical relevance and included (1) core clinical variables (age, gender, sarcoma subtype, primary tumor location, and disease stage at diagnosis), (2) health system factors (initial contact at a specialized sarcoma center (yes/no), number of patient-reported diagnostic barriers), and (3) socioeconomic variables (educational attainment, country income group).

To address missing data in predictor variables, analyses based on multiple imputation were prespecified as the primary analysis. Multiple imputation by chained equations was applied under the assumption of missing at random. Twenty imputed datasets (m = 20) were generated with 20 iterations each. Predictor-specific imputation methods were selected according to variable type, including predictive mean matching for continuous variables, logistic regression for binary variables, polytomous regression for nominal categorical variables, and proportional odds models for ordinal variables. The outcome variable was included as a predictor in all imputation models but was not itself imputed.

As a sensitivity analysis, the same model selection procedure was applied using a complete-case approach, excluding observations with missing predictor data. Results from the complete-case analysis were compared with those from the multiple imputation models to assess the robustness of effect estimates and overall conclusions.

Because the outcome was log-transformed, regression coefficients were exponentiated and reported as the percent change in time to diagnosis, calculated as 100 × (e^β^ − 1). 95% confidence intervals were transformed accordingly. A two-sided *p*-value < 0.05 was considered statistically significant. All analyses were conducted using R (version 4.5.1) [20].

## 3. Results

### 3.1. Characteristics of the Participants

A total of 1803 participants (patients with sarcoma n = 1626 and their caregivers n = 177) completed the questionnaire, provided informed consent and were included in the analysis (Table 1). The median time to diagnosis was 92 days (IQR 31–245 days; min. 0 days, max. 23.25 years), and the median time to first medical care was 30 days (IQR 14–120 days; min. 0 days, max. 19.72 years). Stratified analyses showed similar distributions between patient and caregiver reports, with a median time to medical care of 30 days in both groups and a median time to diagnosis of 92 vs. 90 days. The median age at diagnosis was 53 years (IQR 40–62; min. 0, max. 89).

Most participants were female (1292; 71.7%), while 499 (27.7%) were male. This does not align with the general characteristics of sarcoma, which show a slight overall male predominance [2]. The majority resided in high-income countries (1560; 88.5%), with 130 (7.4%) from upper-middle-income and 73 (4.1%) from low/lower-middle-income countries.

Soft tissue sarcoma was the most common subtype (1069; 59.3%), followed by bone sarcoma (402; 22.3%), gastrointestinal stromal tumors (267; 14.8%), and other sarcoma subtypes (not further defined, 64; 3.6%). The most frequent tumor locations were the lower limb (421; 23.3%), torso/chest (400; 22.2%), and abdomen/pelvis (357; 19.8%). At diagnosis, 1358 patients (75.5%) had localized disease and 394 (21.9%) had advanced or metastatic disease. It is of note that robust data on the proportion of patients presenting with advanced-stage disease are limited, with more recent anecdotal reports indicating up to 40% [21].

The median number of reported barriers was 1 (IQR 1–2). The most frequently reported barriers were misdiagnosis (700; 38.8%) and lack of awareness among providers (610; 33.8%), followed by lack of awareness among patients (492; 27.3%), long waiting times (480; 26.6%), difficulty finding a healthcare provider (476; 26.4%), and financial barriers (143; 7.9%).

### 3.2. Association Between Time to Diagnosis and Patient and Tumor Characteristics

To identify factors associated with prolonged routes to diagnosis in sarcoma, we performed multivariable regression analyses with time to diagnosis as the outcome. In the primary analysis using multiple imputation, patient-reported barriers were strongly associated with longer time to diagnosis, whereas male gender and lower country income level were associated with shorter diagnostic intervals (Table 2).

Each additional reported barrier was associated with a 31.5% increase in time to diagnosis (95% CI 22.0% to 41.6%; *p* < 0.001).

Compared with female patients, male patients had a 40.9% shorter time to diagnosis (95% CI −53.6% to −24.7%; *p* < 0.001). Age was not significantly associated with time to diagnosis (−0.6% per year; 95% CI −1.3% to 0.1%; *p* = 0.115).

Country income group was independently associated with time to diagnosis. Compared with patients from high-income countries, those from low/lower-middle-income countries had a 59.4% shorter time to diagnosis (95% CI −76.0% to −31.5%; *p* < 0.001), and those from upper-middle-income countries had a 41.3% shorter time to diagnosis (95% CI −62.4% to −8.4%; *p* = 0.019).

Sarcoma subtype and stage at diagnosis were not significantly associated with time to diagnosis. Tumor location in the lower limb showed a trend toward longer time to diagnosis compared with abdomen/pelvis (39.4%; 95% CI −0.3% to 94.8%; *p* = 0.052), whereas other locations were not significantly associated with diagnostic interval.

Results from the complete case analysis were consistent in direction and magnitude of effect estimates, confirming the robustness of the primary findings. Overall, health system-related and socioeconomic factors, particularly patient-reported barriers, country income level, and male gender, were the main determinants of time to diagnosis in this cohort.

## 4. Discussion

This study contributes, to our knowledge, the largest international patient- and caregiver-reported survey examining diagnostic experiences in sarcoma. Our findings confirm that timely and accurate diagnosis remains a major challenge across sarcoma subtypes and geographic regions. The median time to definitive diagnosis was 92 days; however, substantial variability in diagnostic intervals was observed. This heterogeneity is consistent with prior literature: A 2020 literature review including 36 studies on soft tissue sarcomas and 34 on bone sarcomas reported wide-ranging diagnostic intervals, spanning from 43.1 to 614.9 weeks in soft tissue sarcomas and from 9 to 120.4 weeks in bone sarcomas, underscoring the pronounced inconsistency in diagnostic trajectories across healthcare settings [22].

Beyond overall prolonged routes to diagnosis, our analysis identified patient-reported barriers, country income level, and gender as independent determinants of diagnostic interval. In contrast, tumor-related characteristics were not independently associated with time to diagnosis.

### 4.1. Patient-Reported Barriers

A central finding of our study is the strong association between patient-reported barriers and prolonged diagnostic intervals. Each additional reported barrier was associated with an increase of more than 30% in time to diagnosis, indicating a cumulative and clinically relevant effect.

Misdiagnosis or prolonged routes to diagnosis was the most frequently reported barrier. This aligns with other literature demonstrating that incorrect or unrevised initial diagnoses substantially prolong diagnostic intervals, particularly when sarcomas are initially assessed as benign lesions or attributed to lifestyle-related causes [16,22]. Misdiagnosis in sarcoma is of particular concern, as it may result in inappropriate treatments, prolonged time to referral to specialist centers, increased risk of recurrence, and potentially inferior oncological outcomes [16].

The findings further illustrate the dual nature of prolonged routes to diagnosis, encompassing both the patient interval (symptom onset to first medical consultation) and the specialist interval (first consultation to definitive diagnosis). Both phases are susceptible to multiple influences. On the patient side, 27.3% of respondents reported insufficient awareness of sarcoma as a barrier. When symptoms are perceived as non-serious, non-urgent, or attributable to benign causes, help-seeking may be delayed [16,22,23]. Younger patients may be more likely to attribute symptoms to benign causes and therefore underestimate their potential clinical significance.

On the healthcare professional side, limited awareness, as well as insufficient sarcoma-specific expertise were frequently reported by respondents (33.8% and 26.4% respectively). Given the rarity and heterogeneity of sarcomas, this is not unexpected. However, it suggests that referral pathways for suspected sarcoma may be unclear or inconsistently applied, particularly in primary care settings where exposure to sarcoma is infrequent. Evidence from prior literature suggests that optimized referral pathways as well as specialized sarcoma care are associated with improved clinical outcomes, emphasizing the need for system-level optimization [24,25].

Beyond tumor biology, patient-experienced and systemic barriers appear to shape the diagnostic trajectory substantially. Strengthening sarcoma awareness, improving transparency around diagnostic and management pathways, and strengthening referral structures may reduce avoidable prolonged routes to diagnosis. Enhanced and early access to accurate patient information could further help timely presentations and navigation through the healthcare system.

### 4.2. Diagnostic Intervals

In the previous literature, median diagnostic intervals in sarcoma typically range from three to six months, with substantial heterogeneity by subtype, healthcare setting, and interval definition. A notable proportion of patients experience longer routes to diagnosis [5,17,26,27]. These estimates are broadly consistent with the intervals observed in our cohort. While the patient interval tends to be longer for most cancer types, sarcoma is notable for having longer primary care intervals, reflecting its ‘hard-to-suspect’ symptom profile. This underscores the need for continued efforts to support the diagnostic process in primary care [28].

Interpretation of these results requires careful consideration of interval definitions. In our study, time to diagnosis was defined as the interval from first symptom onset to confirmed diagnosis. Within the framework of the Aarhus Statement [19], this represents the symptom-to-diagnosis interval rather than the total interval, which extends to treatment initiation. Our approach to assessing time to diagnosis aligns closely with the revised Anderson Model [29], although we combined the “appraisal” and “help-seeking” intervals into a single patient interval and, as noted above, excluded the pre-treatment phase. Further disaggregation into subsequent intervals was not feasible due to the retrospective, self-reported design. This limits inference on the specific stages at which prolonged routes to diagnosis occur.

Furthermore, potential participation bias inherent to survey-based studies should be considered. Selection toward less complex cases cannot be ruled out, particularly if individuals with milder or more clearly symptomatic disease experienced fewer misdiagnoses and shorter intervals. Conversely, survey-based research may overrepresent patients with particularly burdensome or emotionally impactful diagnostic journeys, who may be more motivated to participate in order to highlight systemic challenges.

### 4.3. Sample

In addition, recruitment through patient advocacy networks may preferentially capture individuals with higher health literacy, greater engagement in their care, or more complex diagnostic experiences. This may have contributed to an overestimation of perceived barriers and prolonged routes to diagnosis compared to the broader sarcoma patient population.

The predominance of localized disease among respondents suggests a potential selection bias, with patients with aggressive or rapidly progressive tumors likely under-represented. While robust epidemiological data on stage distribution at diagnosis remain limited, recent reports indicate that up to 40% of patients may present with advanced-stage disease [21], underscoring the likelihood that such patients are not adequately captured in our sample. These individuals may be less able to participate due to clinical deterioration or early mortality, introducing a survivor bias inherent to survey-based designs, in which patients with more favorable disease trajectories are disproportionately represented. At the same time, rapidly progressive tumor biology may, in some cases, prompt expedited diagnostic workup due to acute or severe symptom onset, potentially shortening diagnostic intervals. These opposing mechanisms complicate the interpretation of observed diagnostic timelines. Overall, the underrepresentation of patients with advanced or aggressive disease may influence both the magnitude and direction of observed diagnostic intervals, potentially biasing estimates toward shorter or more homogeneous trajectories. As no data on non-participants are available, the direction and magnitude of potential selection effects cannot be determined. Our findings should therefore be interpreted with caution, particularly when generalizing to the full clinical spectrum of sarcoma and within the context of the heterogeneous literature.

### 4.4. Gender

In our analysis, female gender was independently associated with longer diagnostic intervals. This finding is consistent with findings from the SURVSARC study conducted in the Netherlands, which also identified female gender as a risk factor for prolonged diagnostic intervals based on both clinical and patient-reported data [5]. However, a broad literature review on sarcoma has not consistently confirmed gender as a determinant of prolonged routes to diagnosis [17], indicating ongoing uncertainty regarding its role. Interpretation of this finding requires careful consideration of potential participation bias. The marked predominance of female respondents in our cohort likely does not reflect the true epidemiological distribution of sarcoma but rather differential engagement in survey-based research. Women may be more likely to participate, particularly when recruitment occurs via patient advocacy networks, which may influence observed gender-specific associations. Importantly, our data allow further differentiation of the observed disparity. The patient interval (time from symptom onset to first medical consultation) was identical in women and men (median 30 days), whereas the specialist interval (time from first consultation to definitive diagnosis) was markedly longer in women (median 60 days vs. 35 days in men). These findings suggest that the gender difference in overall diagnostic time is primarily driven by processes occurring within the healthcare system rather than by differences in help-seeking behavior.

Nevertheless, this association should be interpreted with caution. The observed gender effect may partly reflect differences in participation, reporting behavior, or healthcare engagement, rather than exclusively indicating true system-level disparities.

Although the underlying mechanisms cannot be determined from the present data, the findings point toward potential differences in diagnostic evaluation, clinical decision-making, or referral pathways following first presentation. Further research is needed to clarify the structural or clinical factors contributing to this disparity.

### 4.5. Country-Income Level

An unexpected finding was the time to diagnosis in low/lower-middle income countries compared to high-income countries: This seems counterintuitive, as one might expect time to diagnosis to be shorter in high-income countries due to resource disparities in access to general healthcare, pathology services, and structured referral pathways. According to reports from the World Health Organization, pathology services were only available in approximately 35% of low-income countries, compared to more than 95% in high-income countries. In addition, fewer than half of low- and lower-middle-income countries have well-defined referral systems for suspected cancer from primary to secondary and tertiary care [30].

A plausible explanation is selection bias. Patients in lower- and middle-income countries who reach centers more experienced in sarcoma management may represent a selected group with more advanced or symptomatic disease, creating the appearance of shorter diagnostic intervals.

Furthermore, the geographic distribution of our sample is skewed toward high-income countries, with limited representation from low- and lower-middle-income settings. This likely reflects differential access to patient advocacy networks and digital participation, potentially excluding individuals from resource-constrained settings. As a result, the observed income-related differences may partly reflect sampling effects rather than true population-level disparities.

This finding may therefore reflect differential access to specialized care and structured referral pathways rather than intrinsically faster diagnostic processes. Given these considerations, the interpretation of income-related differences in diagnostic intervals should be approached with caution. In the absence of comprehensive population-level data on prolonged routes to diagnosis in rare cancers across income settings, this interpretation remains speculative but consistent with known healthcare inequities.

Participants were drawn from multiple countries with diverse healthcare systems, which may influence diagnostic pathways and patient-reported experiences. Observed prolonged routes to diagnosis likely reflect both local healthcare structures and broader systemic factors. Accordingly, recommendations for healthcare improvement focus on general principles, such as raising awareness, streamlining referral processes, and expanding access to specialized care, while avoiding overgeneralization across specific healthcare systems.

### 4.6. Tumor Characteristics

Tumor-related characteristics including age, sarcoma subtype or stage were not independently associated with time to diagnosis. Only a non-significant associated trend toward a longer diagnostic interval for tumors located in the lower limbs was observed.

This finding is consistent with the biological and clinical heterogeneity of sarcomas, which can occur at any age, in any anatomical location and often present with nonspecific symptoms. Consequently, tumor-intrinsic factors may be less predictive of prolonged routes to diagnosis than patient- and system-level determinants.

### 4.7. Strengths and Limitations

Several limitations should be acknowledged. First, data were self-reported by patients and caregivers and may be subject to recall bias, misclassification, and subjective interpretation of symptoms and healthcare interactions. Independent verification through clinical records was not possible.

Second, the study sample may not be fully representative of the global sarcoma population. Participation was predominantly from high-income countries, with limited representation from low- and lower-middle-income settings, likely reflecting differential access to patient advocacy networks. This may introduce selection effects and requires cautious interpretation of income-related findings.

Third, the predominance of female respondents does not align with the general characteristics of sarcoma, which show a slight overall male predominance [19]. It suggests participation bias and may have influenced observed gender-related associations. In addition, recruitment through patient advocacy networks may preferentially include individuals who are more health-literate, highly engaged, or who experienced more complex diagnostic pathways, potentially leading to overestimation of reported barriers and prolonged routes to diagnosis.

Fourth, the high proportion of patients with localized-stage disease indicates potential survivor bias, as patients with rapidly progressive or fatal disease may be underrepresented, limiting generalizability across the full clinical spectrum.

Finally, the survey did not capture detailed health system characteristics or granular individual-level socioeconomic data. As a result, the analysis of health system and socioeconomic determinants remains limited. Despite these limitations, this study provides important real-world evidence on the diagnostic pathway in sarcoma, an area that remains insufficiently captured in routine clinical datasets. The large international sample enables analysis of patient- and context-related factors across diverse healthcare settings. By systematically assessing diagnostic intervals and patient-reported barriers, this study offers structured insights into processes preceding diagnosis and highlights potential targets for improving the timeliness of care.

## 5. Conclusions

This large international survey highlights that timely and accurate diagnosis of sarcoma remains a substantial global challenge, with considerable variability in diagnostic intervals across subtypes and regions. Beyond overall prolonged routes to diagnosis, our analysis identified patient-reported barriers, country income level, education, and gender as independent determinants of diagnostic interval. In contrast, tumor-related characteristics were not independently associated with time to diagnosis. While detailed health system and individual-level socioeconomic factors were not captured, these findings provide insight into key patient- and context-related factors contributing to prolonged routes to diagnosis. Strengthening sarcoma awareness, optimizing referral pathways, and targeting patient-reported barriers represent actionable strategies to reduce avoidable prolonged routes to diagnosis. Addressing these factors is essential to improving the timeliness of diagnosis and, ultimately, patient outcomes.

Future research should focus on prospective studies with clinical verification, collection of individual-level socioeconomic and health system data, and improved representation of understudied populations to further refine strategies for timely diagnosis.

## Figures and Tables

**Table 1:** Demographic and baseline characteristics of the study population (n = 1803).

Variable	Overall (n = 1803)
Time to diagnosis, days (median [IQR])	92.0 [31.0, 245.0]
Time to medical care, days (median [IQR])	30.0 [14.0, 120.0]
Age, years (median [IQR])	53.0 [40.0, 62.0]
Gender, n (%)	
Female	1292 (71.7)
Male	499 (27.7)
Other	12 (0.7)
Education, n (%)	
Advanced university degree	553 (30.7)
Bachelor’s degree	447 (24.8)
Secondary education	341 (18.9)
Vocational qualification	270 (15.0)
No formal education	57 (3.2)
Other/unspecified	135 (7.5)
Income group, n (%)	
High income	1560 (88.5)
Upper middle income	130 (7.4)
Low/lower middle income	73 (4.1)
Sarcoma type, n (%)	
Bone sarcoma	402 (22.3)
GIST	267 (14.8)
Soft tissue sarcoma	1069 (59.3)
Other	64 (3.6)
Stage, n (%)	
Localized	1358 (75.5)
Advanced/metastatic	394 (21.9)
Unknown	47 (2.6)
Barrier number (median [IQR])	1.0 [1.0, 2.0]
Reported barriers, n (%)	
Difficulty finding healthcare provider	476 (26.4)
Financial barriers	143 (7.9)
Lack of awareness (patient)	492 (27.3)
Lack of awareness (provider)	610 (33.8)
Long waiting times	480 (26.6)
Misdiagnosis	700 (38.8)
Symptoms present, n (%)	1610 (89.3)
Sarcoma center involved, n (%)	978 (56.5)

**Table 2:** Multivariable linear regression analysis of factors associated with time to diagnosis. Complete case and multiple imputation model results.

Variable	Complete Case Model: Effect (%, CI)	*p*-Value	Multiple Imputation Model: Effect (%, CI)	*p*-Value
Age (years)	−0.7% (−1.5 to 0.0)	0.055	−0.6% (−1.3 to 0.1)	0.115
Gender: Female	Reference		Reference	
Male	−42.5% (−55.3 to −26.0)	<0.001	−40.9% (−53.6 to −24.7)	<0.001
Other	−70.4% (−93.4 to 31.9)	0.110	−63.0% (−91.0 to 51.7)	0.167
Sarcoma subtype: Bone sarcoma	Reference		Reference	
GIST	−28.5% (−53.7 to 10.6)	0.132	−30.4% (−54.1 to 5.4)	0.087
Soft tissue sarcoma	−6.7% (−32.3 to 28.7)	0.674	−12.6% (−35.4 to 18.3)	0.383
Other sarcoma	5.6% (−43.3 to 96.6)	0.864	−0.1% (−44.7 to 80.3)	0.996
Tumor location: Abdomen/pelvis	Reference		Reference	
Gynecologic	1.9% (−30.9 to 50.2)	0.925	3.1% (−29.0 to 49.8)	0.872
Lower limb	45.7% (2.8 to 106.4)	0.034	39.4% (−0.3 to 94.8)	0.052
Muscle/skin	10.2% (−65.2 to 249.0)	0.869	1.3% (−66.8 to 209.0)	0.981
Torso/chest	26.0% (−10.5 to 77.5)	0.185	21.8% (−12.1 to 68.7)	0.236
Unknown/multiple	60.8% (−33.1 to 286.8)	0.289	47.9% (−35.0 to 236.6)	0.351
Upper limb	51.7% (−7.4 to 148.5)	0.098	30.7% (−17.8 to 107.8)	0.257
Vascular system	16.9% (−55.0 to 204.1)	0.748	17.7% (−52.0 to 188.4)	0.722
Visceral organs	−10.7% (−57.8 to 89.1)	0.768	−3.8% (−52.6 to 95.3)	0.915
Other	30.1% (−20.6 to 113.1)	0.296	21.6% (−24.0 to 94.5)	0.414
Stage: Advanced	Reference		Reference	
Localized stage	−3.8% (−26.4 to 25.8)	0.780	−1.4% (−23.7 to 27.3)	0.911
Stage unknown	−0.2% (−52.0 to 107.6)	0.997	16.8% (−39.5 to 125.5)	0.643
Number of reported barriers	30.2% (20.4 to 40.9)	<0.001	31.5% (22.0 to 41.6)	<0.001
Income group: High income	Reference		Reference	
Low/lower middle income	−59.6% (−77.0 to −29.0)	0.002	−59.4% (−76.0 to −31.5)	<0.001
Upper middle income	−45.6% (−65.7 to −13.6)	0.010	−41.3% (−62.4 to −8.4)	0.019

## Data Availability

The datasets generated and/or analyzed during the current study are available from the corresponding author upon reasonable request.

## Supplementary Materials

The following supporting information can be downloaded at: https://www.mdpi.com/article/10.3390/cancers18081256/s1, Supplementary Material S1: Global Sarcoma Diagnosis Pathway Survey; Supplementary Material S2: Pilot Testing and Content Validation.

## Abbreviations

The following abbreviations are used in this manuscript:

CI	Confidence interval
IQR	Interquartile range
PI	Patient interval
SI	Care/specialist interval
TDI	Total diagnostic interval
