# Supplementary material for: Factors Associated with Time to Sarcoma Diagnosis: Results from a Global Cross-Sectional Survey by the Sarcoma Patient Advocacy Global Network (SPAGN)
Source: Cancers (Basel). 2026 Apr 16;18(8):1256. doi: 10.3390/cancers18081256 (PMC13115386; doi:10.3390/cancers18081256)
Supplement: Supplementary file 1 [file cancers-18-01256-s001.zip › Suplement S1_SPAGN Diagnosis Survey_Patients_EN_final.pdf]

Global Sarcoma Diagnosis Pathway Survey

For Patients

The importance of early and accurate diagnosis of sarcomas

Early diagnosis of sarcoma can ensure that the tumor is discovered at a time when the disease has not yet spread extensively. This means that the tumor can often be treated less intensively or invasively than if it is diagnosed at a later, more advanced stage.
Moreover, the optimal therapy can only be implemented with an accurate diagnosis. Otherwise, patients are receiving the wrong type or amount (e.g., too much or too little) of tumor therapy.

What we know today

Oftentimes, there is a delay in diagnosing sarcomas. The symptoms for sarcoma are often not identified by the patient nor by a healthcare professional. Additionally, the awareness of sarcomas is low, especially among the first medical contacts. As a result, patients may face delays in being referred to specialists.

What we do not know

Existing data and publications are limited, either to a specific country or region, to single sarcoma subtypes, or detailed questions. But what remains unknown: **What is the global reality of sarcoma patients concerning their diagnosis?**
This is why we are asking for your help: Please share your experiences with us - whether they are positive or negative, both perspectives are important!

How you can help

Your feedback on this survey will help us to analyze the diagnostic process, encompassing the pathway to diagnosis and post-diagnosis management, on a global scale. Based on these results, we aim to work together with you, our specialists, and all stakeholders to **identify the pitfalls and challenges around sarcoma diagnosis.**

What you should know about this survey

- This survey is conducted by patient representatives from the Sarcoma Patient Advocacy Global Network.
- You may participate if you are either a **sarcoma patient, or a caregiver answering on behalf of a sarcoma patient (alive/deceased) or a sarcoma survivor.**
- Your participation in the survey is voluntary
- There are no right or wrong answers to the questions. If you feel uncomfortable answering a question, please choose “I don’t know” or “Prefer not to answer”.
- The survey will take approximately 20 minutes to complete.
- No personal data will be requested from you. This survey is anonymous and resulting data will be published anonymously.
- Should you choose to provide personal or personally identifiable data, we will treat it confidentially. It will not be shared or transmitted to third parties and will be deleted immediately upon receipt.

If you have any questions about the survey, please send them to info@sarcoma-patients.org.

We are very grateful for your cooperation!
The SPAGN Board of Directors & Team

Thank you note

Thank you so much for sharing your experiences with us and for helping us understand the situation around sarcoma diagnosis worldwide!
The SPAGN Board of Directors & Team

Should you choose to provide personal or personally identifiable data, rest assured that we will treat it confidentially. It will not be shared or transmitted to third parties and will be promptly deleted upon receipt.

1. **Are you:**
    - Male
    - Female
    - Non-binary or Transgender
    - Other
    - I prefer not to say

2. **Which of the following best describes the highest level of education you have completed?**
    - No education/primary school
    - Secondary school
    - Qualification in work/business context
    - Bachelor's degree
    - Advanced university degree
    - Other
    - I prefer not to say

3. **Which country do you live in?**
   Dropdown menu countries

4. **Where do you live?**
    - Rural area or town of 10,000 or less
    - Population of > 10,000 – 30,000
    - Population of > 30,000 – 100,000
    - Population of > 100,000 – 500,000
    - Population of > 500,000 – 1 million
    - Population of > 1 million or more
    - I don't know

5. **When were you born?**
    - Date of birth: _____ (Day/Month/Year)

6. **Please state your level of agreement to the statement: General health services and measures are accessible to you.**
    - Strongly agree
    - Somewhat agree
    - Neither agree nor disagree
    - Somewhat disagree
    - Strongly disagree
    - Prefer not to say

7. **Please state your level of agreement to the statement: General health services and measures are affordable to you.**
   - Strongly agree
   - Somewhat agree
   - Neither agree nor disagree
   - Somewhat disagree
   - Strongly disagree
   - Prefer not to say

8. **Please provide the date of each stage:**
   - Date when first symptoms were detected: __________ (Month/Year)
   - Date of first diagnosis: ___________ (Month/Year)
   - Date of current diagnosis: _________ (Month/Year)

9. **How long did it take from the start of your first symptoms to seeking medical care for those symptoms?**
   [Please choose one and fill out in Arabic numerals, e.g. 13, 26, 44]
   - Days: ______________
   - Weeks: ______________
   - Months: ______________
   - The sarcoma was detected by a doctor, no symptoms were experienced before the detection
   - I don't know
   - I prefer not to say

10. **How long did it take from the point when you first contacted a doctor* to when you received the current diagnosis?**
    *This refers to your first contact with a doctor to whom you reported the symptoms of your cancer. Or, if you had no symptoms, it refers to your first contact with a doctor who suspected cancer.
    [Please choose one and fill out in Arabic numerals, e.g. 13, 26, 44]
    - Days: ______________
    - Weeks: ______________
    - Months: ______________
    - I don't know
    - I prefer not to say

11. **What type of sarcoma were you diagnosed with? Please state your current diagnosis.**
    [If the specific subtype is not listed, please choose the general type (e.g., for myxoid liposarcoma, please choose liposarcoma). If the subtype is not listed at all, please choose "other" and specify.]
    - o Ewing's sarcoma
    - o Osteosarcoma
    - o Chordoma
    - o Chondrosarcoma
    - o Alveolar soft part sarcoma
    - o Angiosarcoma
    - o Clear cell sarcoma

- Desmoplastic Small Round Cell Tumor
    - Epithelioid hemangioendothelioma (EHE)
    - Epithelioid sarcoma
    - Endometrial stromal sarcoma
    - Gastrointestinal Stromal Tumor (GIST)
    - Leiomyosarcoma
    - Liposarcoma
    - Malignant peripheral nerve sheath tumor
    - Myxofibrosarcoma
    - Rhabdomyosarcoma
    - Solitary fibrous tumor
    - Synovial sarcoma
    - Sarcoma NOS/Undifferentiated pleomorphic sarcoma
    - Desmoid fibromatosis
    - Tenosynovial Giant Cell Tumor (TGCT)
    - Phyllodes tumor
    - Other subtype, please specify: _______________
- I don't know
- I prefer not to say

12. Did you encounter any challenges or barriers in obtaining a diagnosis of sarcoma? [Select all that apply]

- Difficulty finding a healthcare provider who was knowledgeable about sarcoma
- Long wait times for appointments or test results
- Misdiagnosis or delay in diagnosis
- Lack of awareness about sarcoma among healthcare providers
- Lack of awareness and education on my part
- Financial barriers (cost of medical tests, treatments, etc.)
- Other (please specify)

13. What was the location of the primary tumor you were diagnosed with?

- Upper limb
- Lower limb
- Heart
- Vascular
- Lung
- Head/Neck
- Gynecologic
- Scalp
- Intra-abdominal
- Retroperitoneal
- Torso/pelvic
- Esophagus/throat
- Stomach
- Small intestines
- Rectum
- Spine
- Scull incl. scull base
- Other _________________

- I don't know
- I prefer not to say

14. What stage were you at diagnosis?

- Localized = the tumor had not spread at this time and was limited to one location only
- Advanced/Metastatic = the tumor had already spread to other parts of the body, metastasis was detected
- I don't know
- I prefer not to say

15. Do you or did you have other cancer(s) before the diagnosis with sarcoma?

- Yes, please name diagnosed cancer type: _______
- No
- I don't know
- I prefer not to say

16. Have you been diagnosed with an inherited or genetic syndrome?

- Yes, please specify (FAP, Li-Fraumeni syndrome etc.): __________
- No
- I don't know
- I prefer not to say

17. Which of the following statements best describes your current situation? [Please choose all that apply.]

- Just received a diagnosis / Recently diagnosed
- About to start treatment
- Part-way through the treatment
- In remission, no further treatment needed
- No more treatment is possible
- Refused treatment or further treatment
- Palliative care
- Treatment has been restarted (e.g., due to progression of disease)
- Under regular follow-up (control checkups)
- Out of follow-up (no regular control checkups)
- I don't know
- I prefer not to say
- Other, please specify ___________________________________

18. What were the main symptoms or complications that you experienced before the diagnosis? [Please choose all that apply.]

- Pain
- Lump or swelling
- Fracture
- Loss of mobility
- Weight loss
- Fatigue

- Headaches
- Problems with vision, e.g. double vision
- Fever
- Bruising
- Bleeding
- Nausea
- Bladder / bowel problems
- Paralysis / numbness
- Toothache
- No symptoms, diagnosis was an incidental finding
- I don't know
- I prefer not to say
- Other (please specify) _________________-

Conditional question: If "pain" was chosen

a. **Please describe the pain more specifically. [Please choose all that apply.]**
    - Pain - constant
    - Pain - intermittent
    - Pain worse at night
    - Pain intensifying with time
    - Pain resistant to painkillers
    - I don't know
    - I prefer not to say
    - Other, please describe: __________

Conditional question: If "lump or swelling" was chosen

b. **Please describe the lump/swelling more specifically.** [Please choose all that apply.]
    - Lump painless
    - Lump colorless
    - Lump painful to touch
    - Lump red/angry
    - I don't know
    - I prefer not to say
    - Other, please describe: _________-

Conditional question: If "loss of mobility" was chosen

c. **Please describe the loss of mobility more specifically. [Please choose all that apply.]**
    - Loss of mobility – stiffness
    - Loss of mobility – unable to walk
    - Loss of mobility – bed-bound
    - Loss of mobility – balance
    - Loss of mobility – muscle wasting
    - I don't know
    - I prefer not to say
    - Other, please describe: _________-

19. Has your diagnosis changed in the course of your journey as a patient?

- No

- Yes
- I don't know
- I prefer not to say

Conditional question: If "yes" was chosen

a. **Please state the previous diagnosis:**

- Diagnosed with a different sarcoma subtype – please specify all _______
- Diagnosed with a different condition – please specify all_____________
- I don't know
- I prefer not to say

b. **How many times did the diagnosis change (different condition/sarcoma subtype) before the current sarcoma diagnosis?**
[Please choose one option.]
  - 1
  - 2
  - 3
  - I don't know
  - I prefer not to say
  - More, please specify: __________

20. **Did you receive (any of) the sarcoma diagnosis at a referral / reference / sarcoma specialist center*?**
** A **referral / reference / sarcoma specialist center** is the name for a specialized team for sarcomas – either in one facility or in a network of facilities in different places – which involves all core specialties, draws in other specialties when needed and meets as a multidisciplinary tumor board to decide on therapy strategy.*

- Yes
- No
- This does not apply to my country/healthcare system.
- I don't know
- I prefer not to say

Conditional question: If "yes" was chosen

a. **For which of your diagnosis during your journey have you been referred to a sarcoma center?**
[Please select all that apply]
  - First diagnosis
  - Second diagnosis
  - Third diagnosis
  - I prefer not to say
  - Other, please describe: ____

Conditional question: If "yes" was chosen

b. **How far did you travel to seek advice at a referral / reference / sarcoma specialist center?**
[Please choose one option.]
  - < 100 km/60 miles
  - > 100 km/60 miles – 300 km/190 miles
  - > 300 km/190 miles – 600 km/380 miles
  - > 600 km/380 miles – 1,000 km/620 miles

- More than 1,000 km/620 miles
- I don’t know
- I prefer not to say
- This does not apply to my country/healthcare system. Please explain: ________

21. **Did you seek help in finding a diagnosis outside of your country of residence?**
    - Yes
    - No
    - I don’t know
    - I prefer not to say

    Conditional question: If “yes” was chosen

    a. **In which country did you seek help to find a diagnosis?**
    Dropdown menu countries

    b. **Did you seek help to find a diagnosis in an additional country?**
    Dropdown menu countries

22. **Do you know if any genomic or mutational testing was done for the current sarcoma diagnosis?**
    **Not to be confused with “genetic testing” that looks for genetic mutations that may have been inherited*
    - Yes, genomic testing was done (genomic testing looks broadly for **genomic alterations** in unhealthy/tumor cells)*
    - Yes, mutational testing was done (mutational testing looks for **specific molecular alterations** - point mutations in particular genes)
    - Yes, some testing has been done, but I don’t know what kind
    - No, none was done
    - I don’t know
    - I prefer not to say

    Conditional questions: If option 1-3 (“yes”) was chosen

    a. **When was this testing carried out?**
    - At time of diagnosis
    - Later during treatment
    - Both
    - I don’t know
    - I prefer not to say

    b. **What kind of testing was done?**
    - Whole Genome
    - Exome Sequencing
    - Mutational testing for a specific mutation (e.g., receptor tyrosine kinase KIT or platelet-derived growth factor receptor PDGFR in GIST, NTRK in infantile fibrosarcoma, etc.)
    - I don’t know / I don’t remember
    - I prefer not to say
    - Other, please specify:_____

    c. **Who paid for the test?**
    - me/the patient/the family (out of pocket)
    - The healthcare insurance company

- It was part of a clinical trial
- I don’t know
- I prefer not to say
- Other, please specify: ___________

Oftentimes sarcomas are not recognized immediately by first medical contact persons and patients are referred from one doctor to the other.

23. **On your way to getting a diagnosis, how many doctors did you see for your sarcoma symptoms until your current diagnosis was finally made?**
    Please include healthcare providers of all disciplines (practice-based/hospital) that were consulted, including the medical professional(s) finding the tumor and the one making the final diagnosis.
    In case of more than one sarcoma diagnosis during your journey, please add up all healthcare providers you saw until your current diagnosis.
    [Please choose one option.]
    - 1
    - 2
    - 3
    - 4
    - 5
    - It was an incidental finding and doctor directly suspected sarcoma
    - I don’t know
    - I prefer not to say
    - More, please specify _____________

24. **Which of the following types of doctors did you see prior to the current diagnosis of sarcoma?**
[Please choose all that apply.]
    - General practitioner
    - Internist
    - Surgeon
    - Pediatrician
    - Dermatologist (skin specialist)
    - Gynecologist
    - Orthopedic doctor
    - Physiotherapist/Chiropractor
    - Oncologist
    - Urologist
    - Radiologist (reviewing scans)
    - Emergency/trauma department
    - I don’t know
    - I prefer not to say
    - Other (please specify).____________

25. **What type of doctor first suspected that you may have a sarcoma prior to the current diagnosis?**
    [Please select one response.]
    - General practitioner

- Radiologist (reviewing scans)
- Pathologist (reviewing biopsy/sample)
- Surgeon
- Oncologist
- Pediatrician
- Dermatologist (skin specialist)
- Gynecologist
- Orthopedic doctor
- Physiotherapist/Chiropractor
- Emergency/trauma department
- No doctor suspected it
- I don't know
- I prefer not to say
- Other, please specify: ___________
