# Supplementary material for: Factors Associated with Time to Sarcoma Diagnosis: Results from a Global Cross-Sectional Survey by the Sarcoma Patient Advocacy Global Network (SPAGN)
Source: Cancers (Basel). 2026 Apr 16;18(8):1256. doi: 10.3390/cancers18081256 (PMC13115386; doi:10.3390/cancers18081256)
Supplement: Supplementary file 1 [file cancers-18-01256-s001.zip › Supplement S2_Pilot testing and content validation.pdf]

Supplement S2. Pilot testing and content validation

1. Participants

Pilot testing involved members of the SPAGN network and Board of Directors, including patients, patient advocates, and representatives of sarcoma patient organizations with experience in diagnostic pathways. Participants were recruited through internal SPAGN coordination structures.

2. Procedure

Pilot testing was conducted between April and June 2024 alongside survey development, translation, and technical implementation.

The draft questionnaire was reviewed in multiple rounds by:

- The SPAGN core group and extended team
- International collaborators and member organizations
- Native-speaking contributors involved in translation

Feedback was collected through structured review processes, including iterative review of the English master questionnaire, country-level input during translation, and testing phases during technical implementation.

In addition, the questionnaire was reviewed by a medical oncologist with expertise in sarcoma to ensure medical accuracy and relevance of questions related to the diagnostic pathway.

Testing of translated versions was conducted at country level prior to survey launch to assess clarity, usability, and cultural appropriateness.

3. Assessment of content validity

Content validity was evaluated:

- **Relevance:** assessed through review by patient representatives, international collaborators, and a sarcoma specialist to ensure clinical and experiential appropriateness

- **Comprehensiveness:** evaluated through iterative international feedback to ensure all relevant aspects of the diagnostic pathway were captured

- **Clarity:** assessed through multiple review rounds and multilingual testing to ensure clarity across languages and contexts

4. Results of pilot testing and revisions

Feedback obtained during pilot testing led to refinements of the questionnaire. Key changes included:

Issue identified	Description / Example	Action taken
Clarity	Ambiguity in wording of symptom onset and first consultation	Refined wording and added clarifying descriptions
Completeness	Need to better capture diagnostic intervals and sequence of events	Expanded and structured timeline-related questions
Relevance	Variability of diagnostic pathways across healthcare systems	Adapted items for broader applicability
Response options	Predefined categories insufficient	Expanded response options and added free-text fields

Cultural adaptation	Differences in interpretation across languages	Adjusted wording during translation and review
Technical usability	Issues in navigation and survey logic	Corrected technical implementation

Overall, revisions focused on improving clarity, ensuring comprehensive coverage of the diagnostic pathway, and enabling applicability across diverse international contexts.

5. Translation and language validation

The survey was translated into 19 languages through a coordinated international process involving SPAGN member organizations and native-speaking collaborators.

Translations were based on the English master version and iteratively reviewed to ensure conceptual equivalence, cultural appropriateness, and clarity. Feedback from country representatives was incorporated to resolve ambiguities and adapt wording to local healthcare contexts.

Languages included: English, Hindi, Spanish, Portuguese, German, Dutch, Japanese, Finnish, Swedish, Polish, French, Norwegian, Italian, Hungarian, Russian, Bulgarian, Romanian, and Chinese (two different language versions – simplified/traditional).

6. Finalization of survey

Following iterative pilot testing, translation, and technical validation, the survey was finalized in June 2024 and prepared for international dissemination.
